# Causal relationship between hormone levels and lung cancer: a Mendelian randomization study

**DOI:** 10.3389/fendo.2025.1462531

**Published:** 2025-02-13

**Authors:** Zhiying Feng, Jingmin Fu, Kangyu Wang, Jiaxin Yang, Xuelian Jiang, Qiong Wu

**Affiliations:** ^1^ Department of Traditional Chinese Medicine, College of Traditional Chinese Medicine of Hunan University of Chinese Medicine, Changsha, Hunan, China; ^2^ Department of Humanities and Management, College of Humanities and Management of Hunan University of Chinese Medicine, Changsha, Hunan, China

**Keywords:** Mendelian randomization, hormone levels, lung cancer, lung adenocarcinoma, lung squamous cell carcinoma, small cell carcinoma

## Abstract

**Background:**

Lung cancer is a highly prevalent neoplastic disease in various regions of the world, but the mechanism of its occurrence, development, and metastasis is not clear. Different hormone levels have different potential roles in the occurrence, development, and metastasis of lung cancer, but the association between hormone levels and lung cancer is not clear.

**Objective:**

This study aims to explore the causal relationship between hormone levels and lung cancer using Mendelian randomization. Sensitivity and heterogeneity tests were conducted to ensure the reliability of the results, offering insights into the prevention, diagnosis, and treatment of lung cancer.

**Methods:**

We employed a two-sample Mendelian randomization (MR) analysis using large-scale publicly available genome-wide association studies (GWAS) data to assess the causal relationship between hormone levels and lung cancer. We explored the causal relationship between 15 hormones and three subtypes of lung cancer. The inverse variance weighted (IVW) method was used as the primary analysis, while MR-Egger, weighted median, weighted mode, and simple median were applied as supplementary methods. Sensitivity and heterogeneity tests were conducted to ensure the robustness of the findings.

**Results:**

We identified six hormone levels to be significantly associated with lung squamous cell carcinoma (LUSC): total testosterone, oestradiol, thyrotropin-releasing hormone, insulin, parathyroid hormone, and glucocorticoid. Among them, total testosterone, estradiol, and thyrotropin-releasing hormone were negatively correlated with morbidity. Insulin, prolactin levels, and parathyroid hormone were positively correlated with morbidity. Five hormone levels were significantly associated with lung adenocarcinoma (LUAD): luteinizing hormone, thyroid hormones, insulin, prolactin levels, and parathyroid hormone. Luteinizing hormone and thyroid hormones were negatively correlated with morbidity, while insulin, prolactin levels, and parathyroid hormone were positively correlated with morbidity. Similarly, five hormone levels were linked to small cell lung cancer (SCLC): total testosterone, luteinizing hormone, estradiol, PTHrP, and insulin. Total testosterone and luteinizing hormone were negatively correlated with morbidity, while estradiol, Parathyroid Hormone-Related Peptide (PTHrP), and insulin were positively correlated with morbidity. Several hormones were associated with different subtypes of lung cancer. Insulin was significantly associated with all three types of lung cancer. Testosterone showed positive effects in LUSC and SCLC, and estradiol had varying effects, with a negative correlation in SCLC and a positive correlation in LUSC. Testosterone and estradiol were not significantly associated with LUAD. Luteinizing hormone showed positive effects in LUAD and SCLC, and parathyroid hormone showed negative effects in LUSC and LUAD.

**Conclusion:**

This study demonstrates significant causal relationships between specific hormone levels and various types of lung cancer, providing valuable insights for prevention, diagnosis, and treatment strategies of lung cancer.

## 1 Introduction

Lung cancer, the most common malignancy originating from the trachea, bronchial mucosa, or glands, is classified into three main types: lung adenocarcinoma (LUAD), lung squamous cell carcinoma (LUSC), and small cell carcinoma (SCLC). Globally, lung cancer has the highest incidence and mortality rate among men (1), and its prevalence is on the rise. According to the International Agency for Research on Cancer, lung cancer is one of the leading causes of cancer-related deaths worldwide (2), with 2.2 million new cases and 1.8 million deaths reported in 2020. These account for 11.4% of all new cancer cases and 18.4% of all cancer-related deaths, respectively (3, 4).

Despite advancements in medical technology, the 5-year survival rate for lung cancer remains low at just 18.2%. For advanced non-small cell lung cancer (NSCLC) cases, the survival rate is less than 10%, and for SCLC, the 5-year survival rate is between 5% and 10% (5, 6). Due to non-specific early symptoms and limited screening programs, many lung cancer patients are diagnosed at advanced stages (7), placing a significant economic burden on families and society.

For patients with early-stage NSCLC, surgery is the primary treatment, often supplemented by adjuvant therapy. However, for stage III and IV NSCLC, with the development of medical technology, other treatment methods such as immunotherapy have appeared, but chemotherapy and radiotherapy are still the main treatment methods for lung cancer (8), both of which face significant limitations such as low specificity, poor bioavailability, and the development of drug resistance, which reduces their overall effectiveness (9).

Genetic and environmental risk factors play a crucial role in susceptibility. Hormones, secreted directly into the bloodstream by the endocrine glands, play a critical role in regulating metabolic processes and various physiological functions. Increasing evidence suggests that hormone levels are closely related to lung cancer’s pathophysiological mechanisms and therefore may aid in the prevention, diagnosis, and treatment of this disease (10), for example, in a clinical study, thyroid dysfunction was shown to be an effective predictor of prognosis in patients with non-small cell lung cancer treated with immunotherapy, which included the detection of thyroid-related hormones (11). However, there are relatively few studies examining the effects of various hormones on different types of lung cancer. This paper aims to explore these associations in greater detail, focusing on the relationship between fluctuations in hormone levels and the incidence of LUSC, LUAD, and SCLC.

Over the past few decades, genome-wide association studies (GWAS) have become a vital tool in genetics research. Mendelian randomization (MR) is a statistical method that uses GWAS data to identify single-nucleotide polymorphisms (SNPs) as instrumental variables (IVs) for exposures, such as hormone levels. This approach allows for the investigation of causal relationships between exposures and outcomes (12) while reducing the influence of confounding factors and reverse causation (13).

Using large-scale GWAS data, this study seeks to uncover the potential causal relationships between hormone levels and lung cancer, providing new perspectives on how hormone levels may influence the prevention, diagnosis, and treatment of lung cancer.

## 2 Date and methods

### 2.1 Study design

We used SNPs significantly associated with hormone levels from GWAS data as instrumental variables (IVs), with lung cancer outcomes including LUSC, LUAD, and SCLC. A two-sample Mendelian randomization (MR) analysis was conducted to assess causal relationships (12). The MR framework adheres to three core assumptions: (1) the IV is associated with the exposure, (2) the IV is independent of confounders, and (3) the IV influences the outcome only through the exposure pathway.

### 2.2 Data sources

SNPs associated with hormone levels were selected as instrumental variables (IVs) from publicly available GWAS data, covering 15 hormone categories: testosterone, progesterone, estradiol, luteinizing hormone, prolactin, follicle-stimulating hormone, thyroid hormone, growth hormone, cortisol, thyrotropin-releasing hormone, insulin, parathyroid hormone, glucocorticoid, PTHrP, and corticosteroids. To ensure validity, exposure SNPs had to achieve genome-wide significance (*P* < 5 × 10^-6^) and satisfy linkage disequilibrium (LD) thresholds, with *r* ^2^ = 0.001 and kb = 10,000, in order to minimize potential bias. The GWAS IDs for the LUSC, LUAD, and SCLC datasets were obtained from publicly available GWAS databases.

The statistical data for LUSC, LUAD, and SCLC were accessed from the publicly available GWAS database (https://gwas.mrcieu.ac.uk/). The LUSC GWAS-ID is “ebi-a-GCST004750 “. The LUAD GWAS-ID is “ebi-a-GCST004744 “, and the SCLC GWAS-ID is “finn-b-C3_SCLC “. The LUSC dataset comprised 63,053 individuals, including 55,627 healthy controls and 7,426 patients with LUSC. The dataset contained 7,838,805 SNPs. The LUAD dataset comprised 66,756 individuals, including 55,483 healthy controls and 11,273 patients with LUAD, with a total of 7,849,324 SNPs. The SCLC dataset included 218,792 individuals, comprising 218,613 healthy controls and 1,179 patients with SCLC, and contained 16,380,466 SNPs.

### 2.3 Mendelian randomization analysis

In this study, the inverse variance-weighted (IVW) method, which functions similarly to a meta-analysis, was employed to evaluate the relationships between exposure factors and outcomes (13). A global estimate of the effect of exposure on outcomes was obtained using weighted regression to assess the impact on instrumental variables. The IVW method helps avoid confounding variables that do not exhibit horizontal pleiotropy, thereby producing unbiased estimates. In addition, we applied three **Supplementary Methods**: weighted mode, simple median, and weighted median (12, 14), along with the MR-Egger method (15). If the *P*-value was less than 0.05, the result was considered statistically significant. The *F*-value was set to greater than 10, indicating no weak instrumental variable bias. The formula for calculating the *F*-value is *F* = *R* ^2^(*n* - 2)/(1 - *R* ^2^), where *R* ^2^ represents the proportion of variation explained by SNPs in the exposure dataset, and *N* refers to the GWAS sample size. LUSC, LUAD, and SCLC were treated as dichotomous outcome variables, and their results were expressed in terms of odds ratio (OR) and 95% confidence interval (CI). The analyses were conducted using R 4.3.1 and R Studio software with the “Two Sample MR “ package.

### 2.4 Heterogeneity test, sensitivity analysis, and pleiotropy analysis

Initially, we employed the IVW method to evaluate the correlation between hormone levels and different types of lung cancer, including LUSC, LUAD, and SCLC. MR genetic tools only affect the results when individuals are exposed to them, and gene variants may exert multiple effects. In our primary analysis, we calculated Wald coefficient estimates for each genetic variant and summarized them using the IVW method. The IVW approach, with a multiplicative random effects model, provides a concise estimation while accounting for potential heterogeneity in Wald ratio estimates. However, these estimates may become inaccurate if SNPs affect outcomes through unrelated pathways. To assess heterogeneity, we applied the Cochran Q test. If *P* > 0.05, this indicated no heterogeneity among the SNPs, and a fixed-effects IVW model was used. Conversely, if *P* < 0.05, this suggested heterogeneity among the SNPs, and a random-effects IVW model was applied (16). We also performed a leave-one-out analysis to evaluate sensitivity, systematically removing SNPs one by one to determine if the exclusion of any single SNP significantly impacted the final results. This ensured that the MR results were not disproportionately influenced by any particular SNP. The intercept of the MR-Egger regression test was used to evaluate potential pleiotropy. If *P* > 0.05, it indicated no evidence of pleiotropy.

## 3 Results

### 3.1 Causal relationship between hormone levels and lung squamous cell carcinoma (LUSC)

**Figure 1:**
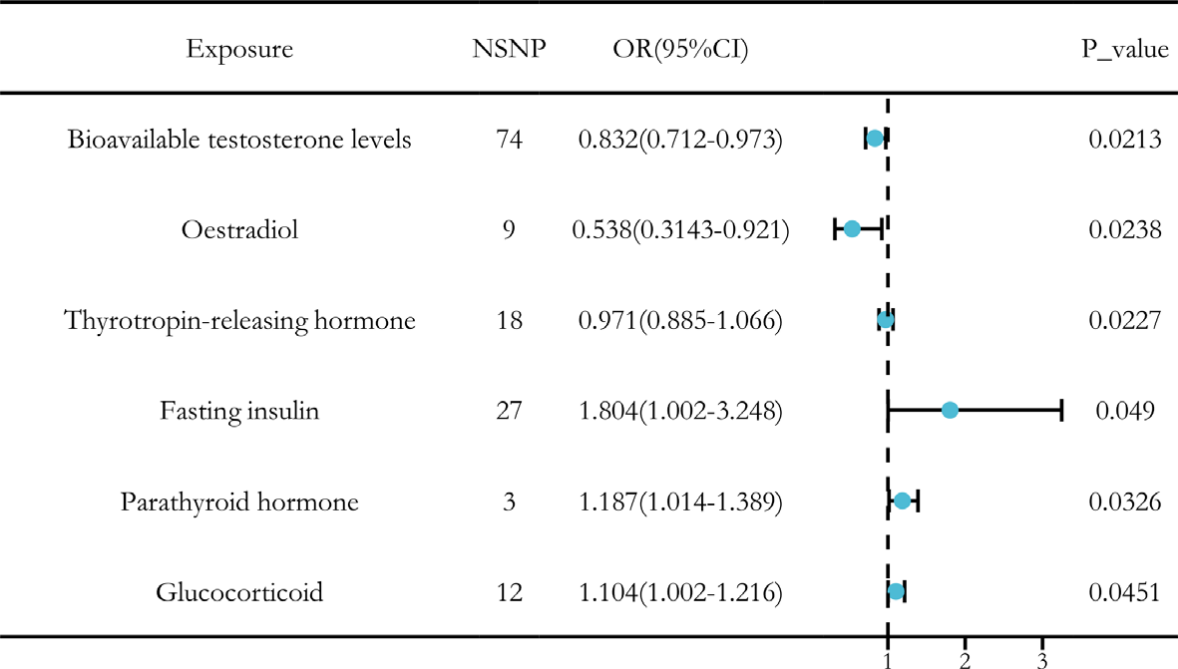
Causal relationships between six hormone levels and LUSC identified through IVW analysis.

After applying a threshold value of *P* < 5 × 10^-6^, we used the IVW method as the primary approach to identify six hormone levels that showed potential causal relationships with LUSC. Among these, three hormone levels were associated with a reduced risk of LUSC: bioavailable testosterone, estradiol, and thyrotropin-releasing hormone. Meanwhile, three other hormone levels were associated with an increased risk of LUSC: fasting insulin, parathyroid hormone, and glucocorticoid (**Figure 1**).

To assess pleiotropy among the instrumental variables, we used the MR-Egger regression method. With the exception of thyrotropin-releasing hormone, no horizontal pleiotropy was detected for the other hormone levels. Additionally, no outliers were found in the outlier analysis. The Cochran Q test was performed to evaluate potential heterogeneity and pleiotropy, and the results indicated no significant heterogeneity or pleiotropy (Q_pval > 0.05). As a result, the fixed-effects IVW model was applied (**Table 1**).

**Table 1:** Results of sensitivity analysis, heterogeneity, and horizontal pleiotropy for hormone levels and LUSC.

Exposure	SNP (n)	MR Egger		Weighted mode		Weighted median		Simple mode		Heterogeneity	MR_harmonize
		OR (95% CI)	P	OR (95% CI)	P	OR (95% CI)	P	OR (95% CI)	P	P	P
Bioavailable testosterone levels	74	0.861 (0.623–1.191)	0.368	0.739 (0.537–1.019)	0.069	0.758 (0.589–0.976)	**0.031**	0.619 (0.381–1.004)	0.056	0.521	0.921
Estradiol	9	1.074 (0.348–3.313)	0.905	0.679 (0.261–1.764)	0.450	0.676 (0.337–1.358)	0.271	0.659 (0.246–1.766)	0.431	0.361	0.942
Thyrotropin-releasing hormone	18	0.755 (0.607–0.940)	**0.023**	0.937 (0.784–1.119)	0.481	0.941 (0.844–1.050)	0.276	0.939 (0.757–1.164)	0.571	0.224	0.683
Fasting insulin	27	0.483 (0.039–6.060)	0.578	1.800 (0.450–7.200)	0.413	1.693 (0.767–3.740)	0.193	1.882 (0.399–8.881)	0.432	0.339	0.950
Parathyroid hormone	3	0.980 (0.617–1.557)	0.945	1.120 (0.882–1.398)	0.452	1.139 (0.928–1.398)	0.214	1.123 (0.881–1.431)	0.448	0.548	0.635
Glucocorticoid	12	1.020 (0.788– 1.319)	0.884	1.024 (0.841–1.247)	0.820	1.057 (0.928–1.204)	0.406	1.016 (0.800–1.291)	0.897	0.391	0.700

The bold values indicated that P < 0.05.

**Figure 2:**
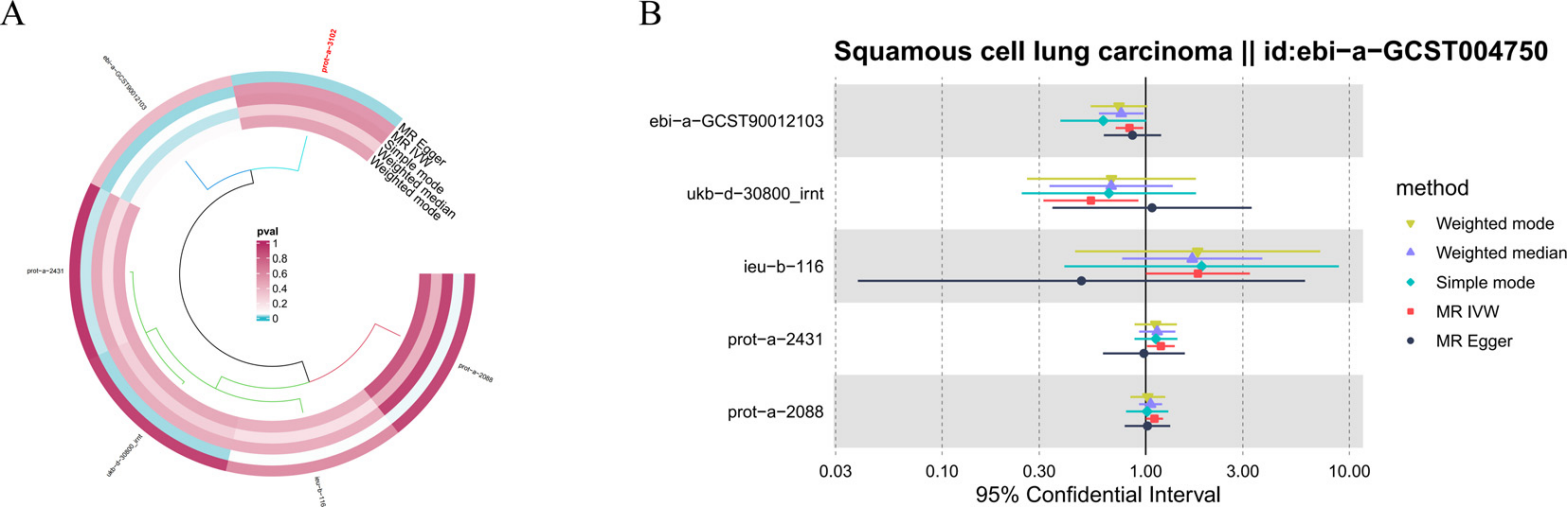
Sensitivity analysis results of hormone levels and LUSC. **(A)** Circular plot for the sensitivity analysis of hormone levels and LUSC. **(B)** The sensitivity analysis results of hormone levels and LUSC include weighted median, MR Egger, and IVW.

In the sensitivity analysis of hormone levels and LUSC, in addition to the IVW method, bioavailable testosterone levels also displayed a *P*-value less than 0.05 in the weighted median analysis, suggesting a significant causal relationship with LUSC. Moreover, the leave-one-out analysis revealed no significant abnormal SNPs across all hormone levels, ensuring the robustness of the findings (**Figure 2**).

**Figure 3:**
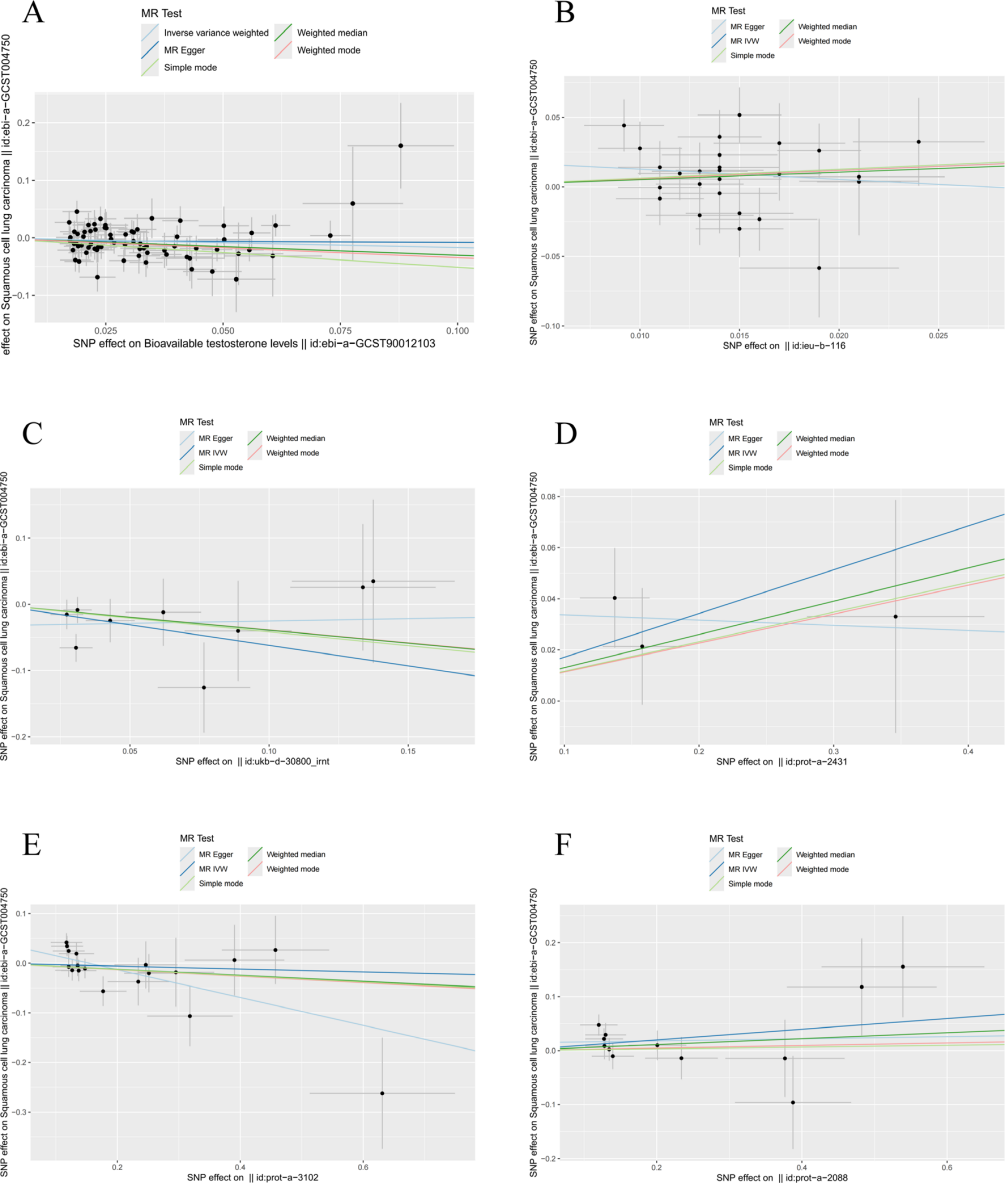
MR analysis of six hormone levels with significant causal associations with LUSC. **(A)** Testosterone, **(B)** Insulin, **(C)** Estradio, **(D)** Parathyroid hormone, **(E)** Thyroid stimulating hormone releasing hormone, **(F)** Glucocorticoid.

In conclusion, we identified significant causal relationships between hormone levels and LUSC, with six hormone levels demonstrating strong associations in five MR analyses with *P* < 0.05. These hormone levels include total testosterone (OR [95% CI] = 0.832 [0.712–0.973], *P* = 0.0236), estradiol (OR [95% CI] = 0.538 [0.3143–0.921], *P* = 0.0238), and thyrotropin-releasing hormone (OR [95% CI] = 0.971 [0.885–1.066], *P* = 0.0227), which were all associated with a reduced risk of LUSC. Conversely, fasting insulin (OR [95% CI] = 1.804 [1.002–3.248], *P* = 0.049), parathyroid hormone (OR [95% CI] = 1.187 [1.014–1.389], *P* = 0.0326), and glucocorticoid (OR [95% CI] = 1.104 [1.002–1.216], *P* = 0.0451) were associated with an increased risk of LUSC (**Figure 3**).

### 3.2 Causal relationship between hormone levels and lung adenocarcinoma (LUAD)

**Figure 4:**
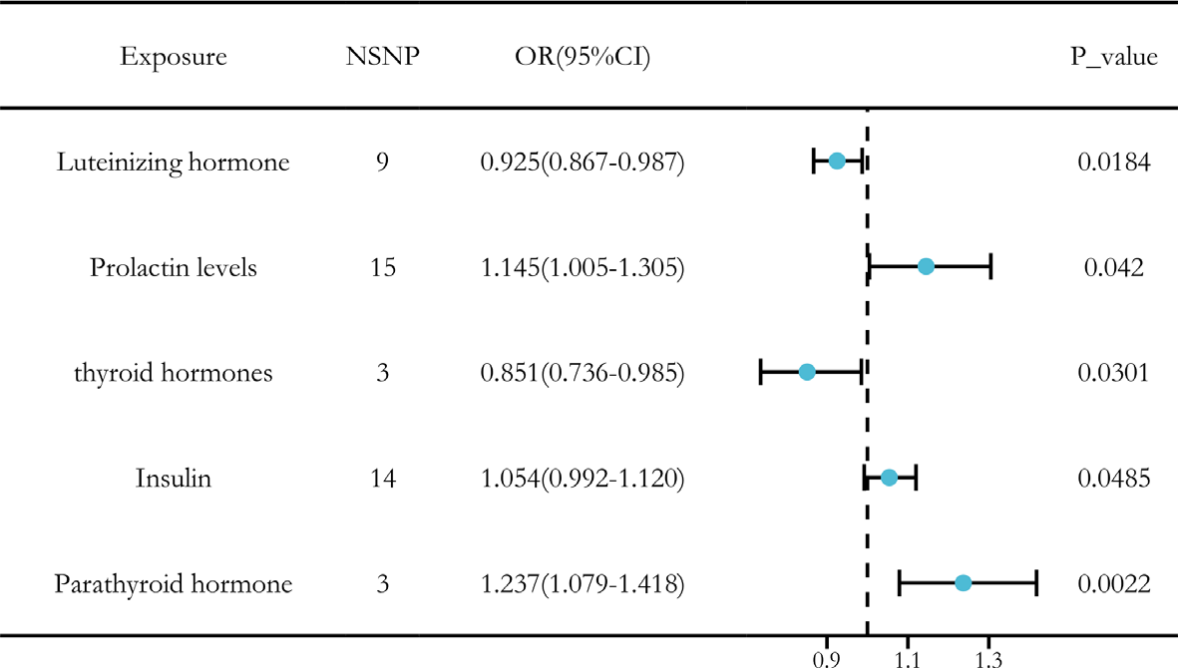
Causal relationships between five hormone levels and LUAD identified through IVW analysis.

After applying a threshold of *P* < 5 × 10^-6^ and using IVW analysis as the primary method, our study identified five hormone levels with potential causal relationships with LUAD. Three hormone levels were associated with an increased risk of LUAD: prolactin, insulin, and parathyroid hormone. Conversely, two hormone levels were linked to a decreased risk of LUAD: luteinizing hormone and thyroid hormones (**Figure 4**).

MR-Egger regression was employed to evaluate horizontal pleiotropy among the instrumental variables, and no evidence of pleiotropy was found for the hormone levels. Additionally, no outliers were detected during the outlier analysis. The Cochran Q test was used to assess both heterogeneity and pleiotropy for the exposure factors, and no significant heterogeneity or pleiotropy was observed (Q_pval > 0.05). As a result, the fixed-effect IVW model was applied (**Table 2**).

**Table 2:** Results of sensitivity analysis, heterogeneity, and horizontal pleiotropy for hormone levels and LUAD.

Exposure	SNP (*n*)	MR Egger		Weighted mode		Weighted median		Simple mode		Heterogeneity	MR _harmonize
		OR (95% CI)	P	OR (95% CI)	P	OR (95% CI)	P	OR (95% CI)	P	P	P
Luteinizing hormone	9	0.902 (0.805–1.010)	0.118	0.905 (0.833–0.984)	**0.047**	0.913 (0.842–0.990)	**0.027**	0.896 (0.779– 1.031)	0.164	0.476	0.630
Prolactin levels	15	0.915 (0.727–1.152)	0.464	1.009 (0.826–1.233)	0.929	1.031 (0.899–1.183)	0.663	1.009 (0.246–1.766)	0.941	0.083	0.892
Thyroid hormones	3	0.798 (0.537–0.1.186)	0.466	0.833 (0.675-1.029)	0.232	0.838 (0.700-1.004)	0.055	0.832 (0.700–1.012)	0.207	0.832	0.667
Insulin	14	1.121 (1.012–1.241)	**0.049**	1.095 (1.027–1.168)	**0.016**	1.094 (1.026–1.167)	**0.006**	1.037 (0.885–1.215)	0.661	0.160	0.642
Parathyroid hormone	3	1.080 (0.732– 1.912)	0.772	1.185 (0.964–1.455)	0.248	1.195 (0.999–1.430)	0.051	1.186 (0.965–1.457)	0.246	0.615	0.635

The bold values indicated that P < 0.05.

**Figure 5:**
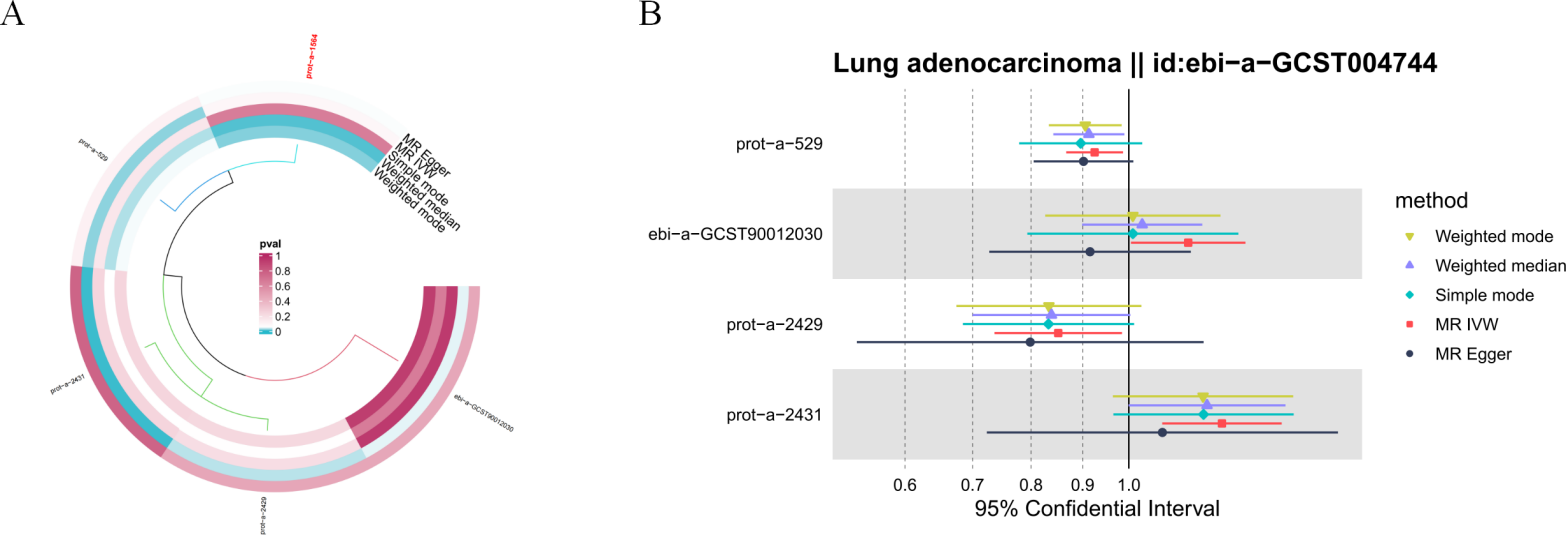
Sensitivity analysis results of hormone levels and LUAD. **(A)** Circular plot for the sensitivity analysis of hormone levels and LUAD. **(B)** The sensitivity analysis results of hormone levels and LUAD include weighted median, MR Egger, and IVW.

**Figure 6:**
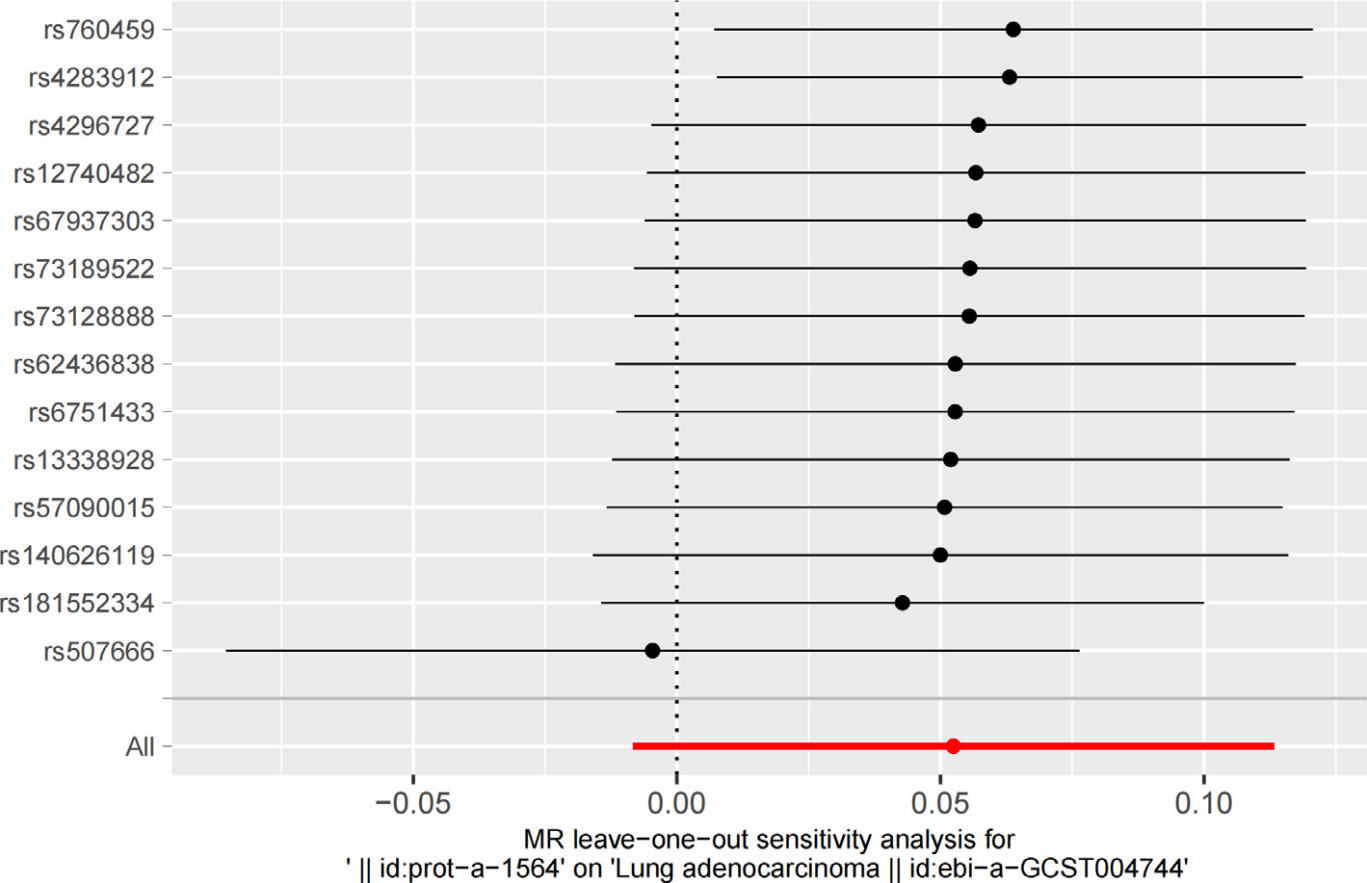
The leave-one-out analysis reveals one individual SNP from hormone levels that have an impact on the outcome.

In the sensitivity analysis for hormone levels and LUAD, beyond the IVW method, both luteinizing hormone and insulin exhibited *P*-values below 0.05 in the weighted median and weighted mode analyses, suggesting a significant causal relationship with LUAD. Moreover, in the leave-one-out analysis, we found that one specific SNP (rs507666) had a considerable influence on the results, particularly affecting insulin (**Figures 5**, **6**).

**Figure 7:**
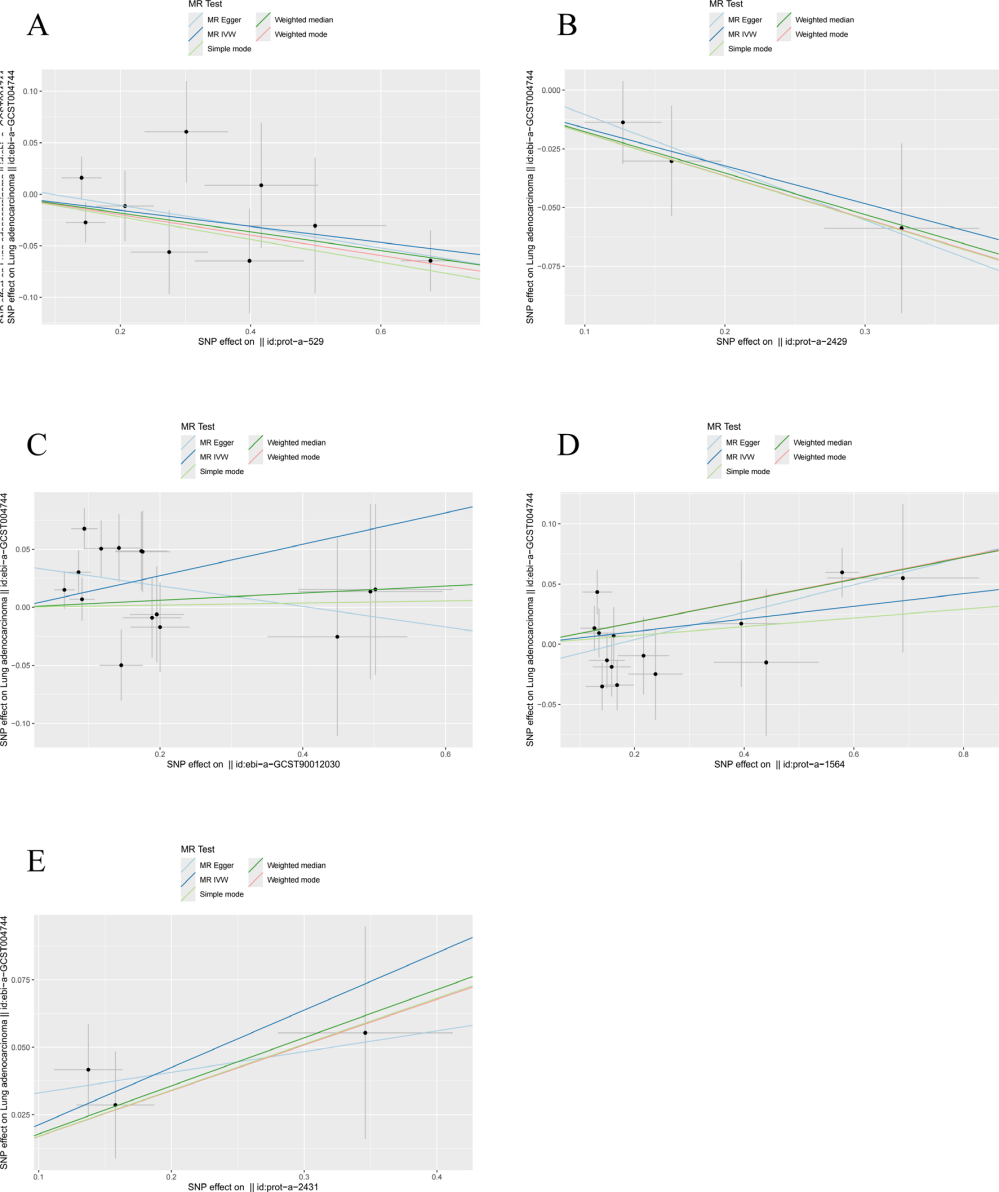
MR analysis of five hormone levels with significant causal associations with LUAD. **(A)** Luteinizing hormone, **(B)** thyroid hormone, **(C)** prolactin, **(D)** insulin, and **(E)** parathyroid hormone.

Ultimately, we identified significant causal relationships between hormone levels and LUAD, with five hormone levels (based on five MR analyses with *P* < 0.05) showing robust associations. These hormone levels include luteinizing hormone (OR [95% CI] = 0.925 [0.867–0.987], *P* = 0.0184) and thyroid hormones (OR [95% CI] = 0.851 [0.736–0.985], *P* = 0.0301), which were associated with a reduced risk of LUAD. On the other hand, insulin (OR [95% CI] = 1.054 [0.992–1.120], *P* = 0.0485) and prolactin (OR [95% CI] = 1.145 [1.005–1.305], *P* = 0.0420) levels and parathyroid hormone (OR [95% CI] = 1.237 [1.079–1.418], *P* = 0.0022) were associated with an increased risk of LUAD (**Figure 7**).

### 3.3 Causal relationship between hormone levels and small cell carcinoma of lung (SCLC)

**Figure 8:**
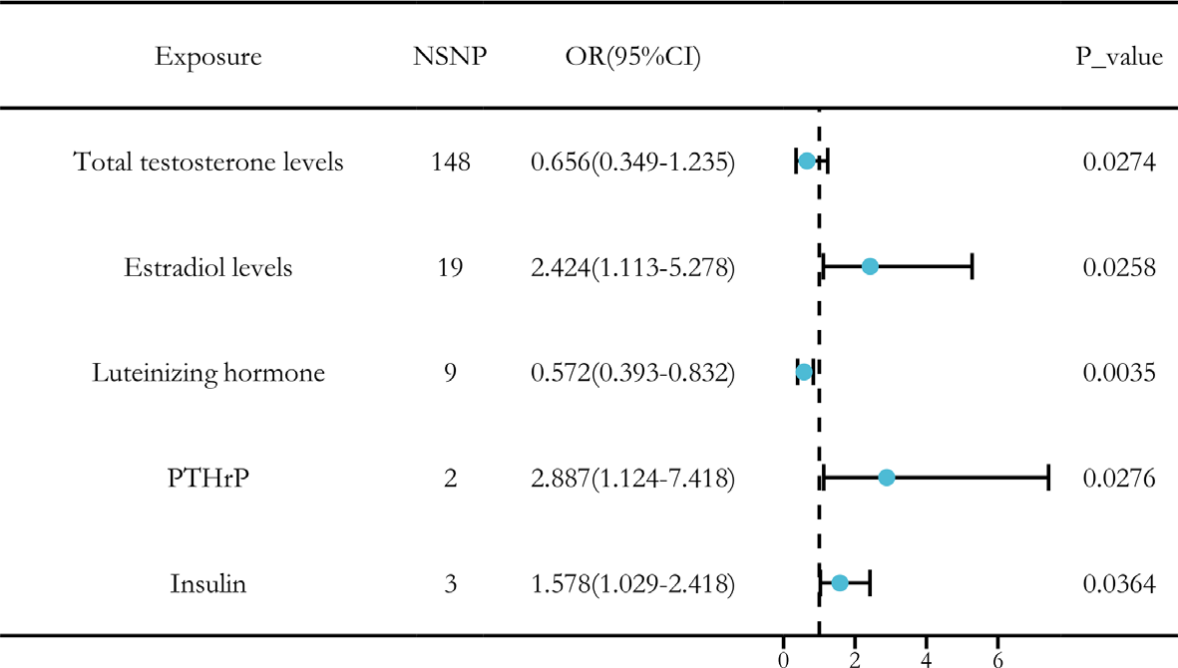
Causal relationships between five hormone levels and SCLC identified through IVW analysis.

After applying the threshold value of *P* < 5 × 10 ^-6^ and using IVW analysis as the primary method, we identified five hormone levels with potential causal relationships with SCLC. Among these, three hormone levels were associated with an increased risk of SCLC: estradiol, parathyroid hormone-related protein (PTHrP), and insulin. Meanwhile, two hormone levels were linked to a decreased risk of SCLC: total testosterone and luteinizing hormone (**Figure 8**).

**Figure 9:**
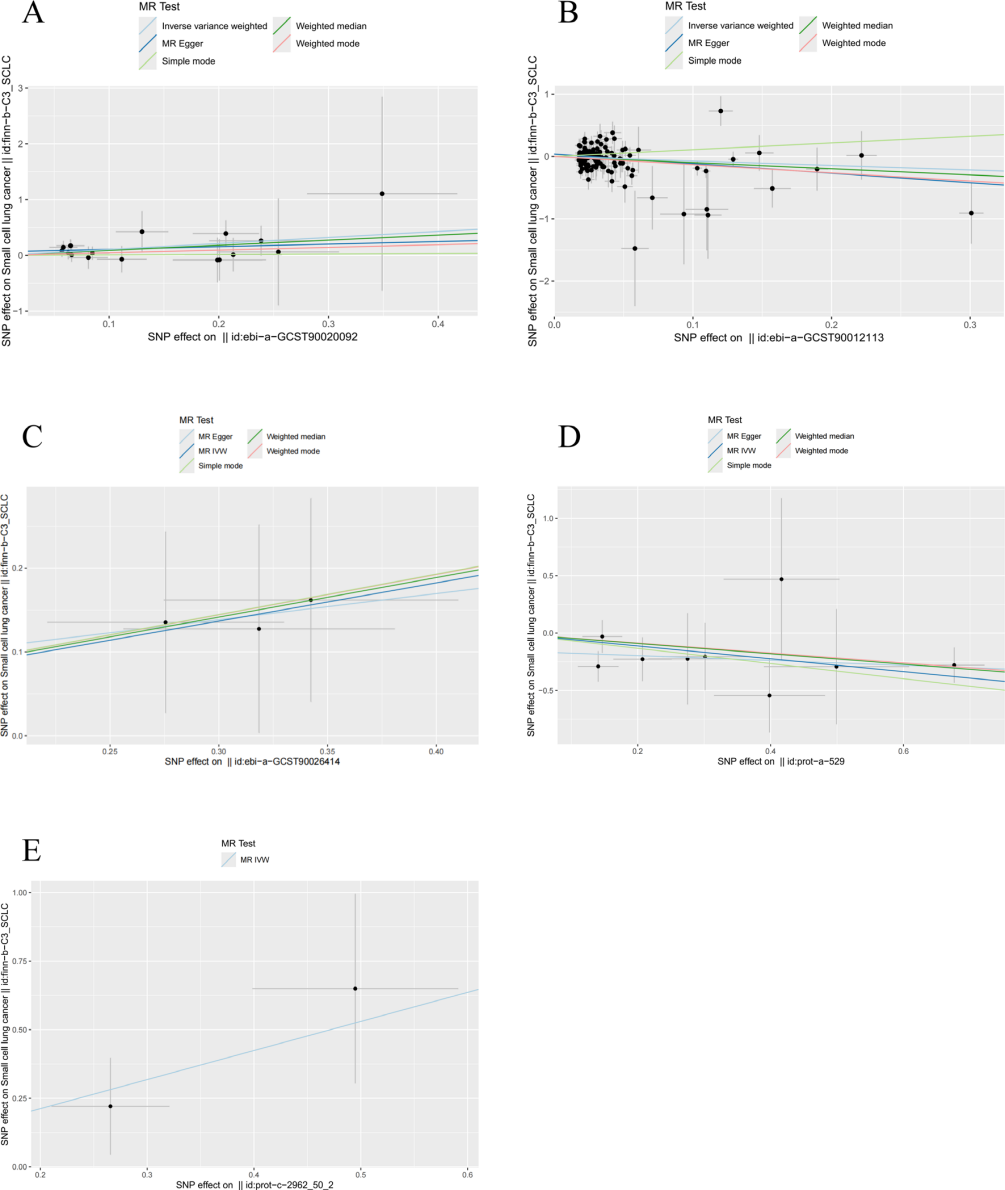
MR analysis of five hormone levels with significant causal associations with SCLC. **(A)** estradiol, **(B)** testosterone, **(C)** insulin, **(D)** luteinizing hormone, and **(E)** thyroid hormone.

Our analysis revealed significant causal relationships between hormone levels and SCLC based on five MR analyses with *P* < 0.05. The hormone levels associated with decreased SCLC risk were total testosterone (OR [95% CI] = 0.656 [0.349–1.235], *P* = 0.0274) and luteinizing hormone (OR [95% CI] = 0.572 [0.393–0.832], *P* = 0.0035). On the other hand, estradiol (OR [95% CI] = 2.424 [1.113–5.278], *P* = 0.0258), PTHrP (OR [95% CI] = 2.887 [1.124–7.418], *P* = 0.0276), and insulin (OR [95% CI] = 1.237 [1.079–1.418], *P* = 0.0364) were significantly associated with an increased risk of SCLC (**Figure 9**).

To assess pleiotropy, we used MR-Egger regression, which found no evidence of horizontal pleiotropy among the hormone levels. Additionally, there were no outliers in the outlier analysis. The Cochran Q test was applied to evaluate heterogeneity and pleiotropy across the exposure factors, and no significant heterogeneity or pleiotropy was detected (Q_pval > 0.05), allowing us to apply the fixed-effect IVW model (**Table 3**).

**Table 3:** Results of sensitivity analysis, heterogeneity, and horizontal pleiotropy for hormones and SCLC.

Exposure	SNP (*n*)	MR Egger		Weighted mode		Weighted median		Simple mode		Heterogeneity	MR_harmonize
		OR (95% CI)	P	OR (95% CI)	P	OR (95% CI)	P	OR (95% CI)	P	P	P
Total testosterone levels	148	0.242 (0.081–0.726)	**0.012**	0.346 (0.123–0.973)	**0.046**	0.614 (0.231–1.635)	0.329	3.575 (0.400– 31.955)	0.256	0.065	0.909
Estradiol levels	19	1.025 (0.203–5.184)	0.976	2.549 (0.565–11.511)	0.239	2.603 (0.880–7.701)	0.084	1.274 (0.221–7.361)	0.789	0.742	0.971
Luteinizing hormone	9	0.810 (0.424–1.547)	0.543	0.649 (0.421–0.999)	0.085	0.637 (0.414–0.981)	**0.041**	0.516 (0.190–1.396)	0.229	0.756	0.628
PTHrP	2	/	/	/	/	/	/	/	/	0.617	0.376
Insulin	3	1.366 (0.013– 143.235)	0.917	1.617 (0.889–2.943)	0.256	1.603 (0.962–2.672)	0.070	1.619 (0.931–2.816)	0.230	0.927	0.686

The bold values indicated that P < 0.05.

**Figure 10:**
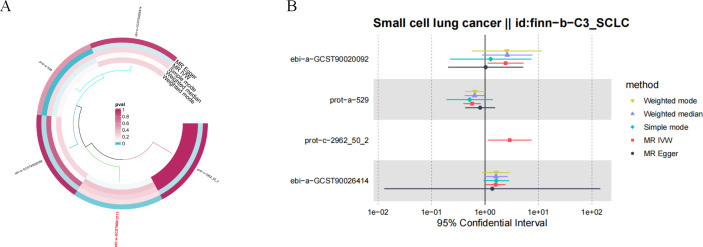
Sensitivity analysis results of hormone levels and SCLC. **(A)** Circular plot for the sensitivity analysis of hormone levels and SCLC. **(B)** The sensitivity analysis results of hormone levels and SCLC include weighted median, MR Egger, and IVW.

In the sensitivity analysis of hormone levels and SCLC, aside from the IVW method, total testosterone showed a *P*-value below 0.05 in the weighted mode analysis, and luteinizing hormone demonstrated a *P*-value below 0.05 in the weighted median analysis, both indicating a significant causal relationship with SCLC. Moreover, the leave-one-out analysis did not identify any statistically significant abnormal SNPs for any of the hormone levels, confirming the robustness of the results (**Figure 10**).

Ultimately, we identified causal relationships between hormone levels and SCLC, with five hormone levels (based on five MR analyses with *P* < 0.05) showing significant associations with SCLC. These hormone levels include total testosterone (OR [95% CI] = 0.656 [0.349–1.235], *P* = 0.0274) and luteinizing hormone (OR [95% CI] = 0.572 [0.393–0.832], *P* = 0.0035), both of which were associated with a decreased risk of SCLC. In contrast, estradiol (OR [95% CI] = 2.424 [1.113–5.278], *P* = 0.0257), parathyroid hormone-related protein (PTHrP) (OR [95% CI] = 2.887 [1.124–7.418], *P* = 0.0276), and insulin (OR [95% CI] = 1.237 [1.079–1.418], *P* = 0.0364) were found to be significantly associated with an increased risk of SCLC (**Figure 9**).

### 3.4 Reverse causal relationship between hormone levels and lung cancer

**Figure 11:**
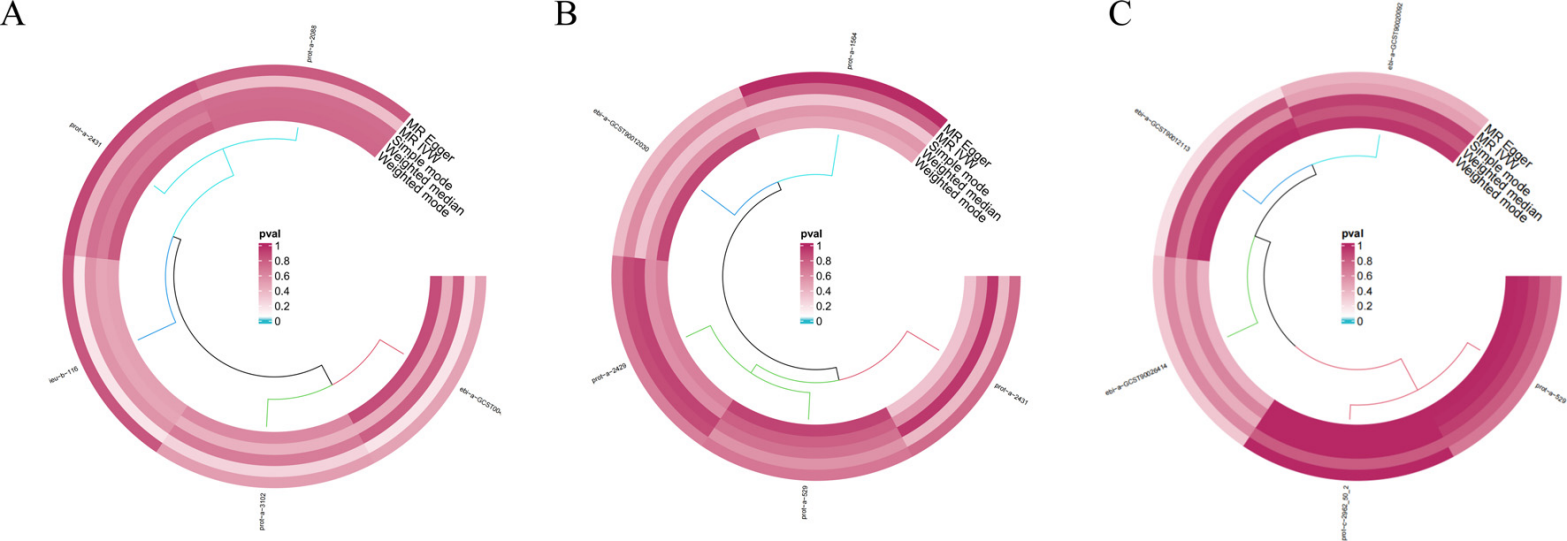
Sensitivity analysis results of the causal relationship between hormone levels and lung cancer. **(A)** Reverse causal relationship between hormone levels and LUSC, **(B)** reverse causal relationship between hormone levels and LUAD, and **(C)** reverse causal relationship between hormone levels and SCLC.

After applying the threshold value of *P* < 5 × 10^-6^ and using multiple methods including MR Egger analysis, IVW analysis, weighted median analysis, simple mode analysis, and weighted mode analysis, we conducted a sensitivity analysis to investigate the reverse causal relationship between hormone levels and lung cancer. Based on five MR analyses with *P* > 0.05, our study found that the reverse causality between hormone levels and lung cancer was not statistically significant (**Figure 11**).

## 4 Discussion

As one of the most prevalent malignant tumors globally, lung cancer is often characterized by non-specific symptoms such as cough and sputum, primarily due to its subtle early-stage manifestations. When more evident symptoms like hemoptysis, dyspnea, chest pain, or hoarseness become apparent, the disease has typically advanced to the middle or late stages (17). Unfortunately, the current early screening and diagnostic methods for lung cancer are not comprehensive enough (18, 19). At the same time, the treatment of lung cancer has been diversified and systematized.

Lung cancer develops in the lungs, which have abundant blood flow, allowing cancer cells to metastasize to various organs through blood transport, with the liver being particularly susceptible (20, 21). The tumor microenvironment plays a crucial role in tumorigenesis, as it comprises tumor cells that interact with surrounding cells via the circulatory and lymphatic systems, thus influencing cancer initiation and progression. Moreover, non-malignant cells within the tumor microenvironment contribute to all stages of carcinogenesis by promoting uncontrolled cell proliferation (22, 23). After the endocrine glands secrete hormones, some of these hormones enter the bloodstream directly. The lungs, as part of the pulmonary circulation, are the route through which various hormones are transported throughout the body via the circulatory system, using blood as the carrier (24).

Hormone levels can affect the early occurrence, middle stage development, and late stage spread of lung cancer through different mechanisms. Hormone levels can affect the development and progression of lung cancer. Studies have shown that lung cancer progression is related to the circadian rhythm of hormone secretion (25). The mechanisms by which hormones affect lung cancer occur through various biological and molecular pathways. Research has demonstrated that estrogen regulates protein kinase by activating the MAPK/ERK and PI3K/AKT pathways, which accelerates lung cancer progression (26). Moreover, 17β-estradiol promotes lung adenocarcinoma progression by upregulating IL6 expression through the ERβ pathway (27). Androgens foster the polarization of M2 macrophages, thereby enhancing the inflammatory environment of the tumor microenvironment, which promotes tumor growth and suppresses anti-tumor immune responses (28). In addition, sex hormones contribute to lung cancer cell proliferation, improve the tumor microenvironment, and promote angiogenesis in lung cancer tissue (29). These studies show that sex hormones promote the occurrence and development of lung cancer by influencing the tumor living environment after the occurrence of lung cancer; especially estrogen plays a major role (30). Insulin has been associated with an elevated risk of lung cancer, as higher fasting serum insulin levels and insulin resistance are correlated with an increased likelihood of developing the disease (31). Our findings align with these results. Additionally, research has shown that nuclear localization of the insulin receptor can promote lung cancer cell proliferation (16). Previous studies using multivariable-adjusted models revealed that free testosterone levels were inversely associated with lung cancer risk, indicating that higher circulating levels of free testosterone may be linked to a lower lung cancer risk (32). However, a prospective study suggested that elevated testosterone levels are associated with 30%–80% higher risk of premature mortality following cancer diagnosis, although they are not linked to the risk of developing new cases of cancer (33). In the microenvironment of non-small cell lung cancer, estrogen has been observed to stimulate cancer cell proliferation and promote tumor growth. Anti-estrogen treatments have shown potential for reducing tumor size, inhibiting growth, and suppressing cell proliferation, potentially improving patient outcomes (34). Another study found that reducing estrogen levels could have a positive impact on the antitumor activity in the affected region (30). These findings are consistent with our results.

Hormone levels not only affect the occurrence and development of lung cancer tissue itself but also affect the metastasis and spread of lung cancer. Some studies suggest that angiogenesis plays a key role in cancer development and metastasis, especially since non-small cell lung cancer is highly vascularized and its progression primarily depends on blood vessel supply (35). Estrogen promotes tumor angiogenesis by activating vascular endothelial growth factor (36) and increasing the production of vascular endothelial growth factor A (37). Luteinizing hormone has been found to inhibit lung cancer cell growth and is used as a treatment for lung cancer (38). Due to insufficient screening programs and the non-specific early symptoms of lung cancer, many patients experience local or distant metastasis, or even advanced disease, at the time of diagnosis (39). Studies have shown that amino PTHrP stimulates cancer progression and accelerates the occurrence and development of lung cancer (40). PTHrP mediates energy expenditure in adipose tissue after cancer and contributes to broader aspects of cancer cachexia (41). Studies have shown that TRH can treat the symptoms of significant fatigue in cancer patients and enhance the body’s immunity (42). In animal models, thyroid hormone T3 inhibits the spontaneous metastasis of lung cancer and prolongs the survival of mice (43).

Hormone therapy has also been shown to have an impact on lung cancer treatment (15). Studies have shown that stress-induced glucocorticoid surges and Tsc22d3 upregulation can interfere with treatment-induced immune surveillance and that glucocorticoid use after immune checkpoint inhibitors may alleviate lung-cancer-related symptoms (44). However, glucocorticoids can act as reactivators in lung cancer development, leading to reversible dormancy in cancer cells. This dormancy is characterized by tolerance to anticancer drugs, activation of survival signals, and increased susceptibility to inhibitors (45). Therefore, hormone therapy for lung cancer is reasonably and cautiously used under the dual verification of basic research and clinical research.

Hormone levels affect the development of lung cancer in multiple dimensions and throughout the disease course. Hormone levels have different effects on the early prognosis of lung cancer, development, and treatment of lung cancer. This effect is related to a number of factors, including gender, genetics, diet, and drug use. It is in this way that the fluctuation of hormone levels has a certain suggestive effect on the treatment and prevention of the deterioration of the early onset of lung cancer—for example, during the treatment of lung cancer, pay attention to the intake of sugar in the diet, female patients in the treatment due to the influence of sex hormones, the effect may have a rebound or not significant phenomenon. The fluctuation of hormone level has a certain suggestive effect on the early onset of lung cancer. In the treatment of lung cancer, some hormones have synergistic or antagonistic effects with drugs. Therefore, in the clinic, we can refer to the changes in hormone levels and make treatment plans. At the same time, in addition to endogenous hormone level fluctuations related to the occurrence of early-onset lung cancer, there are many risk factors for the early occurrence of lung cancer, such as smoking, staying up late, alcoholism, life pressure, etc. In addition to these risk factors related to the occurrence of early-onset lung cancer, they may also affect the fluctuation of hormone levels, thereby promoting the occurrence of early-onset lung cancer. On the whole, fluctuations in hormone levels in the body are associated with early-onset lung cancer and play an important role in the potential occurrence and post-onset of early-onset lung cancer. It has certain guiding significance for doctors in clinical practice. These potential effects need to be further validated with experimental and clinical data.

Our research has several strengths. First, the data on hormone levels and lung cancer came from European populations, avoiding bias related to racial differences and better reflecting the causal relationships between hormone levels and lung cancer. Second, the genetic variation in hormone levels and lung cancer was derived from the largest available GWAS metadata, ensuring the capability of the instrumental variables (IVs) in our MR analysis. Third, our study satisfied all three assumptions of MR analysis. We screened SNPs closely related to hormone levels (*P* < 5 × 10×^-6^) and passed the F-statistical test, satisfying the first assumption. The second assumption was confirmed by removing linkage disequilibrium and evaluating the selected SNPs. Lastly, we used MR-Egger regression to exclude SNPs with horizontal pleiotropy, ensuring that the remaining SNPs met the third assumption. However, there are limitations to our study. First, the conclusions drawn are specific to the European population and may not be applicable to other ethnic groups. Second, the relatively small number of lung cancer cases in the GWAS data introduces the possibility of bias. Lastly, horizontal pleiotropy could not be fully evaluated, even after multiple sensitivity analyses.

## 5 Conclusion

In conclusion, this study assesses the causal effects of hormone levels on lung cancer (including LUSC, LUAD, and SCLC) through two-sample Mendelian randomization (MR) analysis using large-scale, publicly available GWAS data. We evaluated the causal relationships between 15 hormone levels and lung cancer through this method. The analysis identified six hormone levels with significant causal relationships with LUSC, five hormone levels with significant causal associations with LUAD, and five hormone levels linked to SCLC. Our findings demonstrate a strong association between various hormone levels and lung cancer. Insulin was significantly associated with all three types of lung cancer. Testosterone showed positive effects in LUSC and SCLC, and estradiol had varying effects, with a negative correlation in SCLC and a positive correlation in LUSC. Testosterone and estradiol were not significantly associated with LUAD. Luteinizing hormone showed positive effects in LUAD and SCLC, and parathyroid hormone showed negative effects in LUSC and LUAD. This study provides a new understanding of the complex relationship between hormone levels and lung cancer, offering fresh insights into the potential etiology, prevention, diagnosis, and treatment strategies for lung cancer. However, further experimental and clinical research is needed to validate these findings.

## Data Availability

The datasets presented in this study can be found in online repositories. The names of the repository/repositories and accession number(s) can be found in the article/**Supplementary Material**.

## References

[1] Lee CH C S Lee JS H B. Comparison of two meta-analysis methods: inverse-variance-weighted average and weighted sum of Z-scores. Genomics Inform. (2016) 14:173–80. doi: 10.5808/GI.2016.14.4.173 PMC5287121 28154508

[2] Xia C Dong X Li H Cao M Sun D He S. Cancer statistics in China and United States, 2022: profiles, trends, and determinants. Chin Med J. (2022) 135:584–90. doi: 10.1097/CM9.0000000000002108 PMC8920425 35143424

[3] Sung H Ferlay J Siegel RL Laversanne M Soerjomataram I Jemal A. Global cancer statistics 2020: GLOBOCAN estimates of incidence and mortality worldwide for 36 cancers in 185 countries. CA-A Cancer J Clin. (2021) 71:209–49. doi: 10.3322/caac.21660 33538338

[4] Bray F Ferlay J Soerjomataram I Siegel RL Torre LA Jemal A. Global cancer statistics 2018: GLOBOCAN estimates of incidence and mortality worldwide for 36 cancers in 185 countries. CA-A Cancer J Clin. (2018) 68:394–424. doi: 10.3322/caac.21492 30207593

[5] Yin X Li Y Wang H Jia T Wang E Luo Y. Small cell lung cancer transformation: From pathogenesis to treatment. Semin In Cancer Biol. (2022) 86:595–606. doi: 10.1016/j.semcancer.2022.03.006 35276343

[6] Duma N Santana-Davila R Molina JR. Non-small cell lung cancer: epidemiology, screening, diagnosis, and treatment. Mayo Clinic Proc. (2019) 94:1623–40. doi: 10.1016/j.mayocp.2019.01.013 31378236

[7] Herbst RS M D Boshoff C. The biology and management of non-small cell lung cancer. Nature. (2018) 553:446–54. doi: 10.1038/nature25183 29364287

[8] Stárka L Dušková M. What is a hormone. Physiol Res. (2020) 69:S183–5. doi: 10.33549/physiolres PMC8603735 33094616

[9] Ma Z Song P Ji D Zheng M Qiu G Liu Z. Thyroid hormones as biomarkers of lung cancer: a retrospective study. Ann Of Med. (2023) 55:2196088. doi: 10.1080/07853890.2023.2196088 37014291 PMC10075513

[10] Birney E. Mendelian randomization. Cold Spring Harbor Perspect Med. (2022) 12(4):a041302. doi: 10.1101/cshperspect.a041302 PMC9121891 34872952

[11] Zhong X Ying J Liao H Shen L Pan Y. Association of thyroid function abnormality and prognosis in non-small-cell lung cancer patients treated with PD-1 inhibitors. Future Oncol. (2022) 18:2289–300. doi: 10.2217/fon-2021-1537 35440175

[12] Davies NM Holmes MV Davey Smith G. Reading Mendelian randomisation studies: a guide, glossary, and checklist for clinicians. BMJ. (2018) 362:k601. doi: 10.1136/bmj.k601 30002074 PMC6041728

[13] Bowden J Davey Smith G Haycock PC Burgess S. Consistent estimation in Mendelian randomization with some invalid instruments using a weighted median estimator. Genet Epidemiol. (2016) 40:304–14. doi: 10.1002/gepi.2016.40.issue-4 PMC4849733 27061298

[14] Yuan S Kim JH Xu P Wang Z. Causal association between celiac disease and inflammatory bowel disease: A two-sample bidirectional Mendelian randomization study. Front Immunol. (2023) 13:1057253. doi: 10.3389/fimmu.2022.1057253 36685511 PMC9845610

[15] Titan AL He H Lui N Liou D Berry M Shrager JB. The influence of hormone replacement therapy on lung cancer incidence and mortality. J Thorac Cardiovasc Surg. (2020) 159:1546–56.e4. doi: 10.1016/j.jtcvs.2019.10.070 31866083

[16] Qiu R Miao T Lin L Wei Z. Biological functions of nuclear-localized insulin receptor (IR) on A549 lung cancer cells. Endokrynol Pol. (2022) 73:121–30. doi: 10.5603/EP.a2021.0099 34855195

[17] Nasim F Sabath BF Eapen GA. Lung cancer. Med Clinics Of North A. (2019) 103:463–73. doi: 10.1016/j.mcna.2018.12.006 30955514

[18] Skřičková J Nebeský T Kadlec B Hejduk K Májek O Vašáková M. Lung cancer - dia nosis and early detection. Klin Onkol. (2021) 34:6–19. doi: 10.48095/ccko2021S6 34154325

[19] Lovly CM. Expanding horizons for treatment of early-stage lung cancer. N Engl J Med. (2022) 386:2050–1. doi: 10.1056/NEJMe2203330 35403840

[20] Ying X Ma N Zhang X Guo H Liu Y Chen B. Research progress on the molecular mechanisms of hepatic metastasis in lung cancer: a narrative review. Ann Palliat Med. (2021) 10:4806–22. doi: 10.21037/apm-20-1675 33832322

[21] Romaszko AM D A. Multiple primary lung cancer: A literature review. Adv Clin Exp Med. (2018) 27:725–30. doi: 10.17219/acem/68631 29790681

[22] Elhanani O Ben-Uri R Keren L. Spatial profiling technologies illuminate the tumor microenvironment. Cancer Cell. (2023) 41:404–20. doi: 10.1016/j.ccell.2023.01.010 36800999

[23] Arneth B. Tumor microenvironment. Medicina (Kaunas Lithuania). (2019) 56:15. doi: 10.3390/medicina56010015 31906017 PMC7023392

[24] Bahadoran Z M P Azizi F G A. A brief history of modern endocrinology and definitions of a true hormone. Endocrine Metab Immune Disorders-Drug Targets. (2019) 19:1116–21. doi: 10.2174/1871530319666190326142908 30914038

[25] Mazzoccoli G Sothern RB Pazienza V Piepoli A Muscarella LA Giuliani F. Circadian aspects of growth hormone-insulin-like growth factor axis function in patients with lung cancer. Clin Lung Cancer. (2012) 13:68–74. doi: 10.1016/j.cllc.2011.03.034 21729653

[26] Fuentes N Silva Rodriguez M Silveyra P. Role of sex hormones in lung cancer. Exp Biol And Med. (2021) 246:2098–110. doi: 10.1177/15353702211019697 PMC8524770 34080912

[27] Huang Q Zhang Z Liao Y Liu C Fan S Wei X. 17β-estradiol upregulates IL6 expression through the ERβ pathway to promote lung adenocarcinoma progression. J Of Exp Clin Cancer Res. (2018) 37:133. doi: 10.1186/s13046-018-0804-5 29970138 PMC6029357

[28] Becerra-Diaz M Song M Heller N. Androgen and androgen receptors as regulators of monocyte and macrophage biology in the healthy and diseased lung. Front Immunol. (2020) 11:1698. doi: 10.3389/fimmu.2020.01698 32849595 PMC7426504

[29] Dou M Zhu K Fan Z Zhang Y Chen X Zhou X. Reproductive hormones and their receptors may affect lung cancer. Cell Physiol And Biochem. (2017) 44:1425–34. doi: 10.1159/000485538 29186712

[30] Musial C Zaucha R Kuban-Jankowska A Konieczna L Belka M Marino Gammazza A. Plausible role of estrogens in pathogenesis, progression and therapy of lung cancer. Int J Environ Res Public Health. (2021) 18:648. doi: 10.3390/ijerph18020648 33466597 PMC7828659

[31] Argirion I Weinstein SJ Männistö S Albanes D Mondul AM. Serum insulin, glucose, indices of insulin resistance, and risk of lung cancer. Cancer Epidemiol Biomarkers Prev. (2017) 26:1519–24. doi: 10.1158/1055-9965.EPI-17-0293 PMC5626607 28698186

[32] Zhao Y Gao YT Zhang X Rockwood AL Kushnir MM Cai Q. Endogenous sex hormones, aromatase activity and lung cancer risk in postmenopausal never-smoking women. Int J Cancer. (2022) 151:699–707. doi: 10.1002/ijc.34005 35338778 PMC9271581

[33] Ørsted DD Nordestgaard BG Bojesen SE. Plasma testosterone in the general population, cancer prognosis and cancer risk: a prospective cohort study. Ann Oncol. (2014) 25:712–8. doi: 10.1093/annonc/mdt590 PMC4433522 24567517

[34] Smida T Bruno TC Stabile LP. Influence of estrogen on the NSCLC microenvironment: A comprehensive picture and clinical implications. Front Oncol. (2020) 10:137. doi: 10.3389/fonc.2020.00137 32133288 PMC7039860

[35] Daum S Hagen H Naismith E Wolf D Pircher A. The role of anti-angiogenesis in the treatment landscape of non-small cell lung cancer - new combinational approaches and strategies of neovessel inhibition. Front Cell Dev Biol. (2021) 8:610903. doi: 10.3389/fcell.2020.610903 33469537 PMC7813779

[36] Liu C Liao Y Fan S Fu X Xiong J Zhou S. G-protein-coupled estrogen receptor antagonist G15 decreases estrogen-induced development of non-small cell lung cancer. Oncol Res. (2019) 27:283–92. doi: 10.3727/096504017X15035795904677 PMC7848463 28877783

[37] Siegfried JM Gubish CT Rothstein ME Henry C Stabile LP. Combining the multitargeted tyrosine kinase inhibitor vandetanib with the antiestrogen fulvestrant enhances its antitumor effect in non-small cell lung cancer. J Thorac Oncol. (2012) 7:485–95. doi: 10.1097/JTO.0b013e31824177ea PMC3288546 22258476

[38] Abdelbaset-Ismail A Pedziwiatr D Schneider G Niklinski J Charkiewicz R Moniuszko M. Pituitary sex hormones enhance the pro−metastatic potential of human lung cancer cells by downregulating the intracellular expression of heme oxygenase−1. Int J Of Oncol. (2017) 50:317–28. doi: 10.3892/ijo.2016.3787 PMC5182010 27922667

[39] Siegel RL Miller KD Jemal A. Cancer statistics, 2017. CA Cancer J Clin. (2017) 67:7–30. doi: 10.3322/caac.21387 28055103

[40] Hastings RH Montgrain PR Quintana RA Chobrutskiy B Davani A Miyanohara A. Lung carcinoma progression and survival versus amino- and carboxyl-parathyroid hormone-related protein expression. J Cancer Res Clin Oncol. (2017) 143:1395–407. doi: 10.1007/s00432-017-2396-4 PMC11819396 28342003

[41] Kir S White JP Kleiner S Kazak L Cohen P Baracos VE. Tumour-derived PTH-related protein triggers adipose tissue browning and cancer cachexia. Nature. (2014) 513:100–4. doi: 10.1038/nature13528 PMC4224962 25043053

[42] Kamath J Feinn R Winokur A. Thyrotropin-releasing hormone as a treatment for cancer-related fatigue: a randomized controlled study. Supportive Care In Cancer. (2012) 20:1745–53. doi: 10.1007/s00520-011-1268-8 21947558

[43] Deligiorgi MV Trafalis DT. The intriguing thyroid hormones-lung cancer association as exemplification of the thyroid hormones-cancer association: three decades of evolving research. Int J Mol Sci. (2021) 23(1):436. doi: 10.3390/ijms23010436 35008863 PMC8745569

[44] Li N Zheng X Gan J Zhuo T Li X Yang C. Effects of glucocorticoid use on survival of advanced non-small-cell lung cancer patients treated with immune checkpoint inhibitors. Chin Med J. (2023) 136:2562–72. doi: 10.1097/CM9.0000000000002544 PMC10617908 37925595

[45] Prekovic S SK Mayayo-Peralta I. Glucocorticoid receptor triggers a reversible drug-tolerant dormancy state with acquired therapeutic vulnerabilities in lung cancer. Nat Commun. (2021) 12:4360. doi: 10.1038/s41467-021-24537-3 34272384 PMC8285479

